# Purchasing instruments and consumables

**Published:** 2011-12

**Authors:** Ingrid Mason, Phil Hoare

**Affiliations:** CBM Medical Advisor, PO Box 58004, 00200 City Square, Ring Road Parklands, Nairobi, Kenya.; Procurement Manager, Sightsavers; IAPB Procurement Advisor and IAPB Standard List Administrator.

**Figure:**
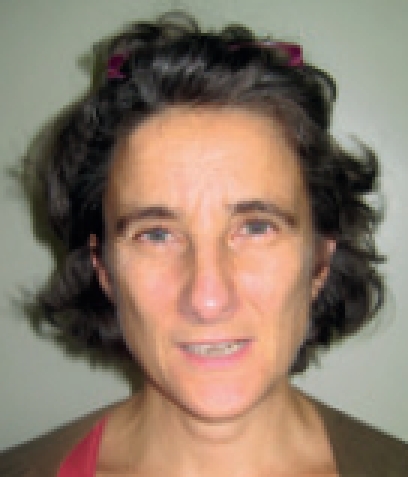
Ingrid Mason

**Figure:**
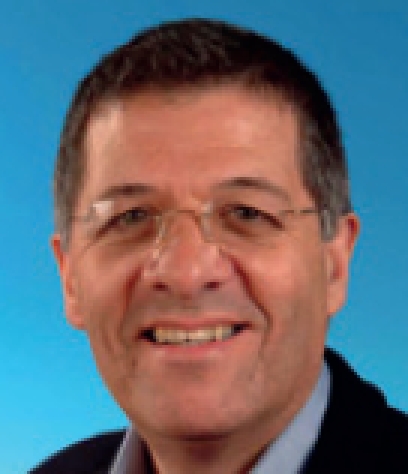
Phil Hoare

**Key learning points**

- Obtain competitive quotes from several reputable suppliers
- When drawing up an order, use the IAPB Standard List for advice on items required, reliable suppliers, and approximate costs
- Where purchasing from outside the country, become familiar with import requirements and which costs must be included in your budget
- Check items carefully on arrival and promptly submit any claims for deficiencies found.

The procedures for purchasing surgical instruments and consumables are similar to those for purchasing equipment, which were addressed in our “Equipment for eye care” issue (number 73, September 2010).

The major difference with consumables is that the number of items in stock (the ‘stock level’) must be monitored constantly. Consumables are generally fast moving and require frequent replacement, which means they must be carefully managed. This is addressed in the article on page 32.

## Drawing up an order

Determine what items are running low, and which you need to re-order. The IAPB Standard List (page 30) provides information on a carefully evaluated range of eye care technologies, supplies, and training materials suitable for use in settings with limited resources. Once the items have been identified, the next stage is to find suitable suppliers and request a quotation.

## Direct local purchase

The first consideration is, are any of the products available locally? If so, do the local suppliers or manufacturers have a good reputation? There may be staff in another eye unit who you could ask; what they have to say about the supplier or manufacturer could save you from an unwise purchase. Has there been a prompt and reliable service, with items priced reasonably? Were the items received of good quality?

**Figure:**
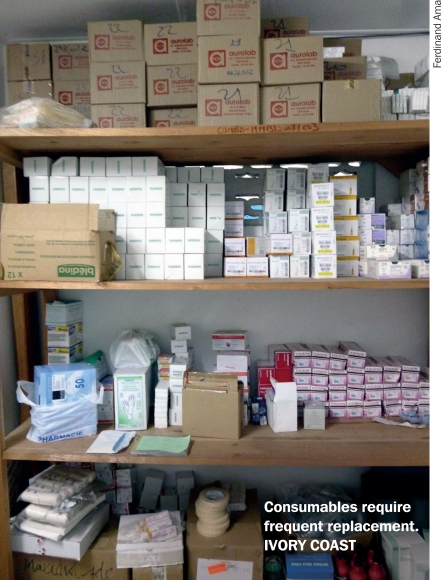


Once you are satisfied that some of the products can be bought locally, you can send a list to the suppliers asking for quotations. It is best to approach more than one supplier to get competitive prices, and to avoid dealing with individual businesspeople.

If you are being offered an instrument or a new brand of consumable, ask for a sample to test to be sure that the quality is of a high standard and appropriate for your needs.

If you have found reliable suppliers and you are happy with the quality of their products, keep a good relationship with them through prompt payment and good communication.

## Using a local agency

In some countries, there are procurement agencies that purchase, stock, and supply government and non-governmental institutions. Working with these agencies, where they are efficient and reputable, can help you maintain quality and ensure that your eye unit always has the necessary items in stock.

The agencies will already have in place the procedures needed to import and clear items through customs. They will also be aware of what can be imported with or without tax exemption, and the national requirements for pharmaceutical products.

Once a good relationship has been built between the procurement agency and your eye unit, provide the agency with a list of the items you use most commonly, and consult the IAPB Standard List for the names of reputable overseas suppliers. This will enable the agency to source your required items on the local market or overseas, and to stock them so that they are always available to your eye unit. Generally, the agency will prefer bulk purchasing as this reduces their costs and they can also negotiate a lower price for the eye unit. Better still, if several eye units can agree to use the same agency, orders can be combined to further lower the price.

**‘If you are being offered an instrument or a new brand of consumable, ask for a sample to test to be sure that the quality is of a high standard’**

## Buying from outside the country

It may be necessary to purchase some of your instruments and consumables from sources outside the country.

Before doing so, you must check what the regulations are for importation. Some countries have websites with information about the regulations but generally it is advisable to appoint a reliable import agent who can guide you through the regulations. There will be a cost to this, so you need to obtain a quotation and allow for these costs in your budget.

Things to consider:

- What are the import duties?
- Are there any restrictions on the products you wish to import?
- Is the manufacturer or country of origin approved by your government?
- Is the manufacturer already registered in your country or does this still need to be completed?
- Do any of the items require a special import licence?
- Does your country stipulate that imported goods have to be inspected before shipment?
- If importing pharmaceuticals, are any of the products deemed controlled substances, such as Ketamine?

There are particular issues around the procurement of pharmaceuticals from out of the country, and many countries have strict regulations. There will be a mandatory registration procedure for the overseas manufacturer and this can be costly. However, not following the procedure will result in penalties. It is therefore important that you check with your ministry of health, particularly if you are considering a new item or a manufacturer you have not used before. A limited selection of eye medicines which have been approved by World Health Organization (WHO) is published in the WHO Model List of Essential Medicines.^1^ The list gives guidance on the basic pharmaceuticals which a country should consider for duty-free import.

When placing an order abroad, you must be familiar with the terms exporters use as part of their contracts. These are known as Incoterms® and were developed by the International Chamber of Commerce.^2^ Incoterms are internationally recognised standards used all over the world. Table 1 shows some of the common terms, together with a brief explanation.

**Figure:**
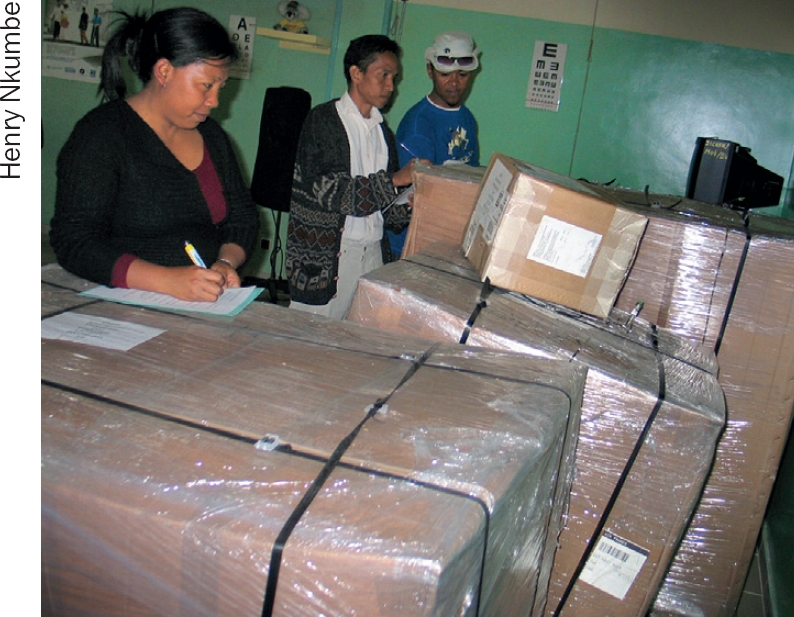
Eye centre staff check goods on arrival. MADAGASCAR

**Table 1.** Incoterms®, the terms commonly used by exporters

**FOB (Free on board)**	This term signifies that the seller is responsible for placing the goods on the ship or plane and that the goods are cleared for export. All other charges, including freight, insurance, and clearance charges on arrival, are the buyer's responsibility.
**CPT (Carriage paid to)**	The seller is responsible for clearing the goods for export, delivering them to the carrier, and onward shipment to the country of destination. The buyer is responsible for insurance and clearance charges.
**CFR (Carriage and freight)**	As for CPT.
**CIP (Carriage, insurance and paid to)**	The seller is responsible for clearing the goods for export, delivering them to the carrier, onward shipment to the country of destination and marine cargo insurance. The buyer is responsible for clearance charges.
**CIF (Carriage, insurance and freight)**	As for CIP.

A few countries require a physical inspection of the goods before shipment, called a pre-shipment inspection (PSI). This must be done by an independent inspection company which checks the goods and verifies the prices, quantities, and quality, as well as the terms of the contract, before permission to import can be granted. If you are placing a large order, it is worth commissioning a PSI as it could well speed up clearance on arrival, but it will increase the cost by about 2–3% of the order. See page 30 for a list of the three largest PSI companies.

Once you have identified your overseas supplier, request a quotation for the items you require. Ask them to include shipment to your country by the most cost-effective method and request marine cargo insurance. The Incoterms° the supplier will use could look as follows: “Total CIF or CIP Nairobi, Kenya: US $5,000.”

If the consignment is small, the supplier may recommend that the products are couriered. This may be ideal, as the consignment will be delivered straight to you, subject to normal import clearance. If the consignment is air freighted or sea freighted, it is the buyer's responsibility to clear the consignment and collect it from the harbour or airport.

To obtain the **total cost** of importing the goods, you must add the following costs to the cost of CIF or CIP shipment to the destination country:

- Import duties
- Import agent's costs
- Inland transportation (from the port of entry to the eye unit)
- Documentation
- Pre-shipment inspection (if required)

If you decide that the price quoted is acceptable, and you have checked each of the items and quantities against your original request, place a confirmed order with the supplier. Ensure that the delivery terms are spelt out, and that you are happy with the shelf life offered for pharmaceuticals (check the shelf life offered before placing the order).

The supplier will either expect payment with the confirmed order or payment prior to despatch. Delays in payment will cause delays in shipment. Monitor the progress of the order with the supplier through regular communication. Ask for full shipping details in advance so that you can put the procedures in place to receive the consignment.

If the consignment is arriving by air you will need the flight and airway bill number and date of arrival. If the consignment arrives by ship, you will need details of the ship, where it is expected to dock, and on what date.

Once received, check the items carefully against your original order. Report immediately any breakages, shortages, or missing items to the manufacturer. If an insurance claim is necessary, there is only a short window of time to submit the claim.

## Safety of medicines and quality of instruments

Counterfeit medicines can be found all over the world. The problem is particularly apparent where governments' regulatory controls are weak, as in some African, Asian, and Latin American countries. Other consumables, such as IOLs, have also been copied illegally. It is therefore important not to accept products unless you are convinced of the reliability of the source.

If purchasing locally, check with the manufacturer or supplier what quality control mechanisms are in place before submitting the order.

The following marks indicate that the products on which they appear are of an acceptable standard: CE Mark, ICO:9001, FDA, and the WHO Good Manufacturing Practices (GMP) certificate.

Cheap instruments of poor quality are not cost-effective, are not appreciated by eye unit staff, and can lead to a poor surgical outcome for the patient.

It is better to procure the highest quality instruments that you can afford. The suppliers and manufacturers listed in the IAPB Standard List have all been tried and tested for the quality of their products, and their prices are affordable.

## Bulk purchasing

Bulk purchasing is about procuring products in larger quantities and transporting them together as one lot. Doing so reduces the cost of transport and therefore the individual cost of each item.

For imported instruments and consumables, bulk purchasing items in frequent use will significantly reduce costs.

A supplier may well be prepared to offer large discounts, so consider placing an order covering a period of three or six months at a time. Hospitals managed by the same religious institution or supported by the same NGO in a country or region could consider combining their orders. A discussion within the National Prevention of Blindness or VISION 2020 Committee could lead to a joint order, which will be cost-saving for all those involved, and also reduce the amount of time and effort spent on clearing customs.

In competitive markets, or where there are local suppliers, it may not be necessary to bulk buy as you could negotiate a discount based on your projected requirements over a year or six months (see page 34).

**FROM THE FIELD: Setting up and managing a depot for instruments and consumables**

**Figure:**
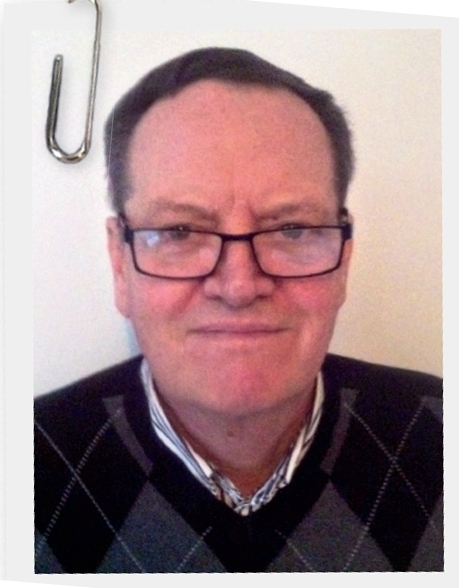
**Paul Caswell**: CBM Country Co-ordinator, Nigeria

In countries where foreign exchange or importation are difficult and where there is low but constant demand from a number of small eye units, a depot for instruments and consumables can make the difference between the success or failure of a VISION 2020 programme.

In Nigeria, there are many small eye care programmes, often in remote areas. The CBM country co-ordination office identified the need for a depot to provide a constant supply of essential instruments and consumables. It was started with a grant from CBM, but now all running costs are generated by the sales of the items stocked.

Ideally, a depot should be a one-stop shop. We try to carry everything that an eye care programme needs: consumables (eye drops, IOLs, viscoelastic, sutures etc); instruments, and equipment, such as operating microscopes. The depot also carries popular textbooks, public awareness materials, and information about training.

Although our depot is not expected to make a profit, the selling price of each item must not only cover the cost price, insurance and freight, clearance charges, and local transport, but must be high enough to contribute to the running costs of the depot. Running costs include staff salaries, maintenance, utilities, administration, and auditing. There should even be a little left to cover inflation.

When starting the depot, we were visited by ‘businessmen’ coming disguised as eye care centre representatives. They would buy our goods at our lower prices and then sell them on at inflated prices to eye care programmes that did not know of our existence. However, as our reputation grew, this problem has disappeared.

If you plan to start an eye care depot, it is good to situate it near a large town, such as the capital city. Eye care personnel are likely to visit the capital a few times each year, so that the visit to the depot will be just one extra task. We also agree to despatch goods to our customers by courier service, after receiving a deposit in our account.

**Figure:**
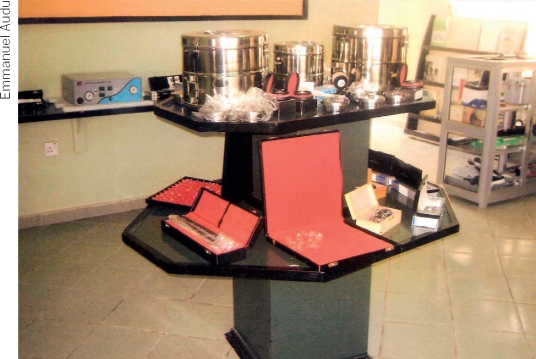
Some of the eye care equipment offered for sale in the depot. NIGERIA

**‘Ideally, a depot should be a one-stop shop’**

One of the most critical factors in starting a depot in Nigeria was identifying an appropriate person to manage it. We felt that an experienced sales manager was needed rather than a specialist in eye care. This has proved to be a good decision. The manager quickly learnt about eye care equipment and consumables by reading catalogues and talking to customers about their needs. He attended a short course about procurement but otherwise had no formal, specialist training. If customers have questions he cannot answer, he is always willing to ask advice from his ophthalmologist colleagues in the unit to which the depot is attached.

To run a depot is not easy. It requires a lot of administration: ordering, banking, auditing, and so on. However, I am convinced it has an important part to play in making VISION 2020 a reality.

**CBM Resource Centre**

Maryland-Gwagwalada, Abuja, Tel: + 234 (0) 805 232-2411, Email: cbm_rc@yahoo.com
